# Production of Ramoplanin and Ramoplanin Analogs by Actinomycetes

**DOI:** 10.3389/fmicb.2017.00343

**Published:** 2017-03-06

**Authors:** Mercedes de la Cruz, Ignacio González, Craig A. Parish, Russell Onishi, José R. Tormo, Jesús Martín, Fernando Peláez, Debbie Zink, Noureddine El Aouad, Fernando Reyes, Olga Genilloud, Francisca Vicente

**Affiliations:** ^1^Fundación MEDINA, Centro de Excelencia en Investigación de Medicamentos Innovadores en AndalucíaGranada, Spain; ^2^Merck Research Laboratories, MerckKenilworth, NJ, USA; ^3^Biotechnology Programme, Spanish National Cancer Research CentreMadrid, Spain; ^4^Centre des Sciences et Techniques, Université Ibn ZohrAgadir, Morocco

**Keywords:** high-throughput screening, microbial natural products, antibacterial activity, actinomycetes, ramoplanin, peptidoglycan intermediate Lipid II

## Abstract

Ramoplanin is a glycolipodepsipeptide antibiotic obtained from fermentation of *Actinoplanes* sp. ATCC 33076 that exhibits activity against clinically important multi-drug-resistant, Gram-positive pathogens including vancomycin-resistant *Enterococcus* (VRE), methicillin-resistant *Staphylococcus aureus* (MRSA), and vancomycin-intermediate resistant *Clostridium difficile*. It disrupts bacterial cell wall through a unique mechanism of action by sequestering the peptidoglycan intermediate Lipid II and therefore does not show cross-resistance with other antibiotics. However, while demonstrating excellent antimicrobial activity in systemic use in animal models of infection, ramoplanin presents low local tolerability when injected intravenously. As a consequence of this limitation, new derivatives are desirable to overcome this issue. During a natural product screening program developed to discover compounds that disrupt bacterial cell wall synthesis by inhibiting peptidoglycan transglycosylation through binding to the intermediate Lipid II, 49 actinomycete strains were identified by HR-LCMS as producers of ramoplanin-related compounds. The producing strains were isolated from environmental samples collected worldwide comprising both tropical and temperate areas. To assess the diversity of this microbial population, the 49 isolates were initially identified to the genus level on the basis of their micromorphology, and 16S sequencing confirmed the initial identification of the strains. These analyses resulted in the identification of members of genus *Streptomyces*, as well as representatives of the families *Micromonosporaceae, Nocardiaceae, Thermomonosporaceae*, and *Pseudonocardiaceae*, suggesting that the production of ramoplanins is relatively widespread among Actinomycetes. In addition, all of these isolates were tested against a panel of Gram-positive and Gram-negative bacteria, filamentous fungi, and yeast in order to further characterize their antimicrobial properties. This work describes the diversity of actinomycete strains that produced ramoplanin-related compounds, and the analysis of the antimicrobial activity exhibited by these isolates. Our results strongly suggest the presence of new ramoplanin-analogs among these actinomycete producers.

## Introduction

New antibiotics with new mechanisms of action are urgently needed because clinically significant bacterial pathogens have not only acquired resistance to nearly all existing antibiotics, but also increasingly exhibit multi-drug resistance (Gerits et al., 2016). Considering that the present antibiotic therapies have become increasingly inefficient, new technologies are required to identify and develop novel classes of antibacterial agents.

While searching for novel antibacterial leads, an attractive alternative to the classical target-based approach is the use of promoter-inducible reporter assays that are amenable to high-throughput screening. These assay systems are based on cells that carry reporters such as β-galactosidase or luciferase genes fused to promoters that specifically respond to certain types of antibiotic stress (Shapiro and Baneyx, 2002; Sun et al., 2002; Fischer et al., 2004). Those genetically modified strains are particularly advantageous for high-throughput assay systems considering the low compound concentrations that they require. The selective induction of the reporter fusion indicates that a compound is at least perturbing the pathway of interest as part of its biochemical impact (Fischer et al., 2004).

DNA microarray analysis of the response of *Bacillus subtilis* treated with sublethal concentrations of the cell wall antibiotic bacitracin revealed the presence of overlapping regulons controlled by alternative sigma factors and two component systems. One operon strongly induced by bacitracin, which was identified by John Helmann's laboratory at Cornell University, contains the genes *lia*IHGFSR (*liaRS* stands for lipid II cycle interfering antibiotic response Regulator and Sensor). The β-galactosidase gene *lac*Z was placed downstream of the promoter for this operon (P_*liaI*_) such that induction of the operon by bacitracin or other compounds that have the same effect on the liaRS two component regulatory system will induce the production of β-galactosidase. This can then be monitored as an indication of antibiotic action that is impacting this liaRS system (Cao et al., 2002; Mascher et al., 2003).

Lipid II is a membrane-bound cell wall precursor which performs the cycle of peptidoglycan “building block” translocation. The efficiency of targeting Lipid II as an antibacterial strategy is highlighted by the fact that it is the target for at least four different classes of antibiotics (lantibiotics, mannopeptimycins, ramoplanin, and vancomycin). Since this pathway is limited to prokaryotes and low toxicity is expected, it is highly desirable to discover novel structural classes that interfere with it. Moreover, the emergent problem of bacterial resistance to drugs such as vancomycin has led to increasing interest in the therapeutic potential of other compounds that target Lipid II (Donadio et al., 2002).

A natural product screening program was developed to detect inhibitors that interfere with stage II peptidoglycan biosynthesis. This screening approach called “LiaRS assay” allowed the identification of positive controls such as ramoplanin.

Ramoplanin is a lipoglycodepsipeptide antibiotic derived from *Actinoplanes* spp. ATCC 33076 and was first isolated as a complex of three closely related components A1, A2, and A3 (A1, 6–12%; A2 72–86%; A3 8–14%) in which A2 was the most abundant (Cavalleri et al., 1984). The structural difference is in the length of the N-terminal acyl chain. Ramoplanin A2 exhibits activity against clinically important Gram-positive bacteria including vancomycin-resistant *Enterococcus* sp. (VRE), methicillin-resistant *S. aureus* (MRSA) and vancomycin-intermediate resistant *Clostridium difficile* (Finegold et al., 2004; Peláez et al., 2005). Preclinical studies have also demonstrated that ramoplanin exerts a rapid bactericidal effect on *S. aureus* biofilms (Schmidt et al., 2010) and that a clinical vancomycin-resistant *S. aureus* strain containing the vanA gene was susceptible to ramoplanin (Bozdogan et al., 2003). Recently, it has been reported (by Nanotherapeutics) to have additional activity against *C. difficile* spores, both *in vitro* and in an animal model (Jabes et al., 2014). Ramoplanins have a unique mechanism of action that disrupts bacterial cell wall by interfering with late-stage transglycosylation cross-linking reactions in peptidoglycan biosynthesis. Ramoplanin A2 acts by sequestering the Lipid Intermediate II, which keeps this substrate from accurate use in downstream reactions catalyzed by transglycosylases that produce mature peptidoglycan polymer. This antibiotic works at a site complementary to vancomycin and shows no cross-resistance with other glycopeptides. At present, ramoplanin is being developed for the targeted prophylaxis of recently treated patients with *C. difficile* infection (CDI) at high risk for infection relapse. Twelve Phase I studies, two Phase II studies (one in CDI and one in VRE) as well as one Phase III study (in VRE) have been conducted (http://www.nanotherapeutics.com/ramoplanin/). Although Phase III study was not completed.

Results presented in this paper illustrate that this screening approach allowed us to detect actinomycete strains that produced ramoplanin-related components. The diversity of those actinomycete strains is described, along with the analysis of the antimicrobial activity exhibited by these isolates. Likewise, HR-LCMS analyses strongly suggest the presence of new ramoplanin-analogs among these actinomycete producers and current large scale fermentations and purifications are being performed for the identification of these novel antimicrobials. To our knowledge, this is the first report that the production of ramoplanin-type natural products is relatively widespread within Actinomycetes.

## Materials and methods

### Morphological identification of actinomycete strains

Actinomycetes were tentatively identified to the genus or family level after direct observation of the microscopic morphology (400 × and 1000 × magnification with long distance range objectives) of the vegetative and aerial mycelium and characteristic sporulating structures developed upon growth on water agar for 21 days at 28°C (Goodfellow et al., 1984, 2012).

### DNA extraction and molecular characterization

Total genomic DNA from the actinomycetes used in this study was purified as previously described (Innis et al., 1990) from strains grown in ATCC-2 liquid medium [0.5% yeast extract (Difco, Franklin Lakes, NJ, USA), 0.3% beef extract (Difco), 0.5% peptone (Difco), 0.1% dextrose (Difco), 0.2% starch from potato (Panreac, Barcelona, Spain), 0.1% CaCO3 (E. Merck, Darmstadt, Germany), and 0.5% NZ amine E (Sigma, St Louis, MO, USA)]. PCR primers fD1 and rP2 were used for amplifying the nearly full-length 16S ribosomal RNA genes of the strains (Weisburg et al., 1991). PCR products were sent to Secugen (http://www.secugen.es/) for sequencing, and were used as a template in sequencing reactions using the primers fD1 and rP2, and 1100R and 926F (Lane, 1991). Partial sequences were assembled and edited using the Assembler contig editor component of Bionumerics (ver 5.10) analysis software (Applied Maths NV, Sint-Martens-Latem, Belgium).

The identification of the closest match sequences was performed against the database of type strains with validly published prokaryotic names (Chun and Int, 2007) which was implemented at the EzTaxon server (http://ezbiocloud.net/eztaxon; Kim et al., 2012).

### Characterization of actinomycete strains

The soil samples used for the isolation of the 49 actinomycete strains were collected worldwide comprising both tropical and temperate areas: Costa Rica, French Guyana, Mexico, New Caledonia, South Africa, Spain, and Sri Lanka, including different ecological habitats like agricultural soils, riverbeds, lakes, ponds, swamps, dunes, tropical and sempervirent forests, savanna soil, and rhizospheres.

The strains were tentatively identified on the basis of their macro and micro-morphology and the genus assignment of 39 strains was confirmed by 16S rDNA gene sequencing. The most abundant taxonomic group was the family Micromonosporaceae (71.4%) with 34 strains identified as members of the genus Micromonospora and 1 strain of Actinoplanes. The remaining strains were, identified as Nocardia (3 strains), Streptomyces (3 strains), Actinomadura (1 strain), Amycolatopsis (1 strain), and Lechevalieria (1 strain). Five strains could not be assigned to any genus and were morphologically identified as filamenting Actinobacteria (**Table 2**).

### Natural products extract library generation

Bacteria and fungi were grown in complex liquid media containing different amounts of nutrient sources: carbon sources (e.g., monosaccharides, disaccharides), complex carbon sources (e.g., wheat flour, soluble starch), nitrogen sources, and mineral salts sources (R. Tormo et al., 2003). The secondary metabolites in the broths were extracted with acetone (1:1) and shaking in an orbital shaker for 1 h. The extracts were then centrifuged at 1,500 × g for 15 min. 1.5 mL of DMSO were added to aliquots of 15 mL and the resulting mixture was evaporated in a Turbovap equipment until half of the volume remained, ~7.5 mL, to a final crude concentration of 1xWBE (whole broth equivalents) and 20% DMSO. Five-hundred microliters of these crude extracts were stored at −20°C in 96-well ABgene plates until needed.

### LiaRS screening assay

The screening assay was performed against two *Bacillus subtilis* strains. The lacZ gene was fused to the liaI promoter (P liaI) in both strains such that induction of this promoter results in the production of β-galactosidase. One strain contains the lacZ fusion and additionally has a kanamycin insertion into the liaR gene that inactivates liar. The P liaI-lacZ fusions were licensed from Cornell University by Merck & Co., Inc. (Mascher et al., 2003, 2004). Strains belong to the Merck culture collection with the following codes: HB0950 (MB5826, CU1065 SPb P-liaI74-cat-lacZ) and HB0953 (MB5827, CU1065 SPb P-liaI74-cat-lacZ liaR::kan). Afterwards, Merck & Co., Inc. donated both of them to Foundation MEDINA. Strain HB0953 contains the lacZ fusion but additionally has a kanamycin insertion into the liaR gene which inactivates liaR. *B. subtilis* strain HB09050 or HB09053 was incubated to stationary phase in LB (1% NaCl) at 37°C and 220 rpm. The culture was adjusted to an optical density of 0.25 at a wavelength of 600 nm. Then, 100-mL of LB agar (1% NaCl) that contained 250 μg/ml of X-gal was inoculated with 1 mL of bacteria. The inoculated agar was poured into a NUNC bioassay plate (245 × 245 mm). After the agar solidified, the plates were dried in a laminar-flow hood for 15 min. After that, 0.02 mL drops of natural product extract were placed on the surface of the agar and the plates were allowed to dry in a laminar-flow hood for 15 min. The plates were incubated for 18 h at 37°C. They were scored for zones of inhibition and for hydrolysis of X-gal (blue precipitate). Bacitracin (50 mg/mL) was used as positive control of the assay while vancomycin (0.5 mg/mL) was used as negative control.

### *In vitro* antimicrobial activity

#### MIC determination assays

MICs values of samples against a panel of strains from MEDINA collection which are: *Staphylococcus aureus* (CL 860), *Haemophillus influenzae* (CLB 21526), *Pseudomonas aeruginosa* (PAO-1), *Escherichia coli* (ATCC 25922), *Enterococcus faecalis* (ATCC 29212), and *Candida albicans* (MY 1055) strains were determined by the microdilution technique in accordance with the guidelines of the National Committee for Clinical Laboratory Standards (NCCLS, 2007) using inocula of 1 × 10^5^–5 × 10^5^ CFU/mL. Serial dilutions of extracts were performed to assess growth inhibition. Microtiter plates were incubated at 35°C for 20–24 h. Cell growth was monitored by comparing the optical density between the time of treatment and after incubation time. The lowest concentration causing 90% inhibition microbial growth was defined as the MIC.

#### Aspergillus fumigatus agar-based assay

The *A. fumigatus* (MF5668: ATCC13073) stock conidial suspension was adjusted by quantitative colony counts at 3.5 × 10^9^ CFU/mL. The conidial suspension was diluted into Yeast Nitrogen Base broth (YNB, 6.75 g L^−1^ yeast nitrogen base) to 65% transmittance at 660 nm. Then, 10 mL of this inoculum broth was added to 1 L of Yeast Nitrogen Base-Dextrose and 20 mL of the seeded agar media were poured into Omnitray plates. Once the agar solidified, the plates were dried in a laminar flow hood for 15 min. After this time 10 μL of each extract were distributed on the surface of agar plates and incubated at 29°C for 18 h. Finally, the halos of inhibition were measured. Amphotericin B (0.25 mg/mL) was used as a positive control for this assay.

### Macromolecular labeling assay

The labeling reaction contained 0.05 mL of a log phase culture of *Bacillus subtilis* HB0950 in nutrient broth that contained 1% NaCl, 0.025 mL of two-fold concentrated nutrient broth (2% NaCl) that contained 7.5 mCi/L of L-[2, 3-^3^H]-aspartic acid, 5 mCi/L of [2-^14^C]-thymidine, 100 mg/L of chloramphenicol or 0.8 mg/L of rifampicin and 0.025 mL of inhibitor (1xWBE). The samples were incubated 37°C on a microtiter plate shaker. After 30 min, 0.025 mL of 25% TCA was added to stop radioactive incorporation. The TCA-insoluble material was collected on a glass microfiber filtermat with a Skatron Cell Harvester. The filtermat was washed with distilled H_2_O and dried under a stream of hot air. Radioactivity was measured in a Betaplate Scintillation Counter.

### Liquid chromatography-high resolution mass spectrometry and data analysis

Two micro liters of the extracts were analyzed by LC-HRMS. Analysis was performed on an Agilent (Santa Clara, CA) 1100 single Quadrupole LC-MS, using a Zorbax SB-C8 column (2.1 × 30 mm), maintained at 40°C and with a flow rate of 300 ul/min. Solvent A consisted of 10% acetronitrile and 90% water with 0.01% trifluoroacetic acid and 1.3 mM ammonium formate, while solvent B was 90% acetronitrile and 10% water with 0.01% trifluoroacetic acid and 1.3 mM ammonium formate. The gradient started at 10% B and went to 100% B in 6 min, kept at 100% B for 2 min, and returned to 10% B for 2 min to initialize the system. Full diode array UV scans from 100 to 900 nm were collected in 4 nm steps at 0.25 s/scan.

HRMS data was acquired in a Thermo Finnigan LTQ-FT with the standard Ion Max API source (without the sweep cone) and ESI probe. Three scan events were used. The ion trap was scanned from 150 to 2,000 first in negative ion mode and then in positive ion mode. The FT was scanned from 200 to 2,000 in the positive ion mode only. In all cases the SID was set to 18 volts to try to reduce multiple ion clusters.

Data were analyzed with Apex software. The standard conditions were; peak width/resolution is set to 100,000 resolution at mass 400, MS search tolerance set between 0.008 and 0.003 Da. Apex software is a product from Sierra Analytics (Modesto, CA).

## Results

### Validation of LiaRS assay

**Figure 1:**
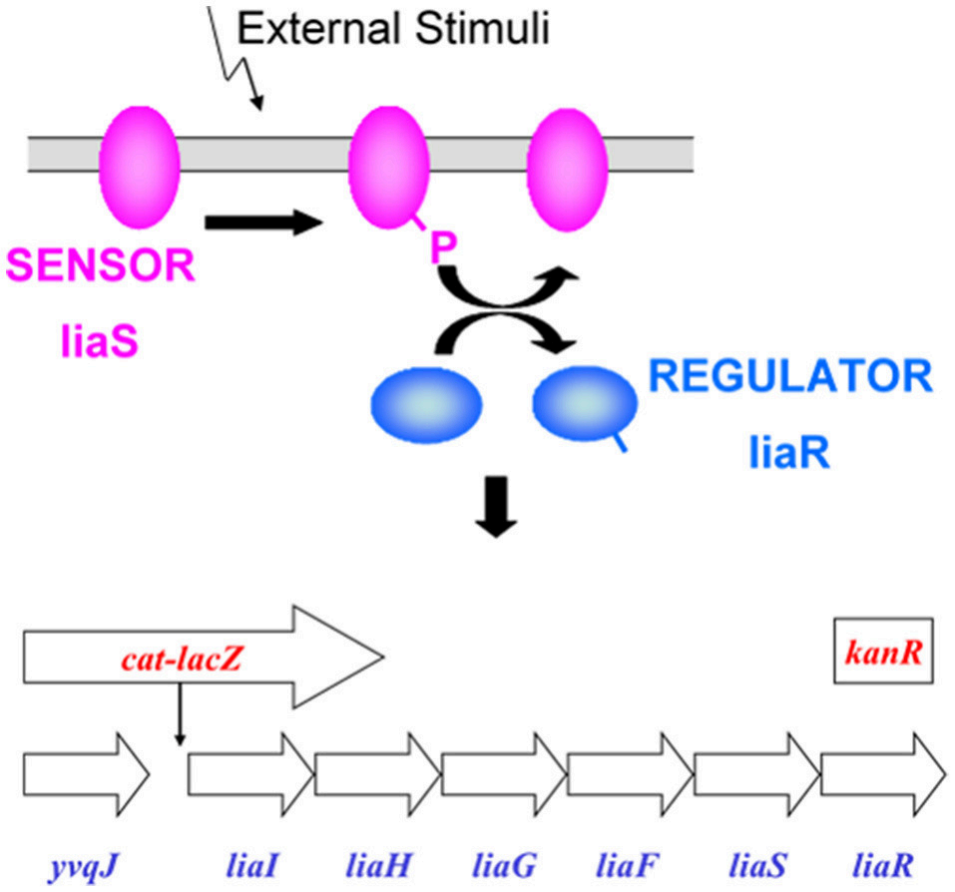
**LiaRS—Lipid II cycle interfering antibiotic response Regulator and Sensor: Two-component antibiotic sensing system in *Bacillus subtilis* that co-ordinates genetic response to inhibitors of peptidoglycan synthesis that interfere with the lipid II cycle**. *Background strain*: *Bacillus subtilis* CU1065; *HB0950*: lacZ fusion; *HB0953*: lacZ fusion, kanR insertion (liaR gene disrupted). LiaRS autoregulates the *liaIHGFSR* operon. Induction of the *liaIH* operon mediated by the two-component *liaRS* sensor/regulator system. Specificity associated with liaRS. *lacZ* inserted downstream from P_liaI_. Induction results in production of β-galactosidase. Induction of β-galactosidase in *B. subtilis* HB0953 indicates that induction was not due to an inhibitor of peptidoglycan synthesis.

The LiaRS (Lipid II cycle interfering antibiotic response Regulator and Sensor) two-component system (Figure 1) is one of several antibiotic-sensing systems that coordinate the genetic response to cell wall active antibiotics. Upon addition of inhibitors of stage II peptidoglycan synthesis, LiaRS autoregulates the liaIHGFSR operon. The lia promoter responds strongly and specifically to antibiotics that interfere with the lipid II cycle. The induction of β-galactosidase by these types of antibiotics is detected with X-gal, which turns blue when hydrolyzed by β-galactosidase. It is possible to differentiate specific interference of the lipid II cycle because specific inhibitors will induce the production of β-galactosidase in strain HB0950 but not in HB0953, in which the *liaR* gene has been disrupted. The LiaRS system and the two *Bacillus* strains have been previously described by Mascher et al. (2004).

*B. subtilis* HB0950 (MB5826, CU1065 SPb P-liaI74-cat-lacZ) produces β-galactosidase in response to the induction of liaI by certain cell-wall active antibiotics. *B. subtilis* HB0953 (MB5827, CU1065 SPb P-liaI74-cat-lacZ liaR::kan) is a liaR knock-out mutant. It is also capable of synthesizing β-galactosidase, but not in response to the liaIHGFSR operon.

In *B. subtilis* HB0950, the lacZ gene has been fused to the liaI promoter (PliaI) such that induction of this promoter results in the production of β-galactosidase. This production can be detected when this strain is grown on Luria agar (LA) medium containing either 5-bromo-4-chloro-3-indolyl-β-D-galactoside (X-gal) or 3,4-cyclohexenoesculetin-β-D-galactopyranoside (S-gal) in the presence of an inducer. Detection of β-galactosidase activity in HB0950 has been shown to be 150–200-fold over background in the presence of bacitracin, ramoplanin, and nisin, and ~35-fold over background in the presence of vancomycin (Mascher et al., 2004). Other antibiotics affecting protein synthesis (chloramphenicol, kanamycin, spectinomycin, streptomycin, tetracycline), RNA synthesis (rifampicin), and peptidoglycan synthesis (β-lactams, moenomycin, D-cycloserine), did not induce β-galactosidase production; neither did detergents (SDS, Triton) nor uncouplers of oxidative phosphorylation (DNP, CCCP). The production of β-galactosidase by HB0950 appears to be a specific response to compounds that bind to or otherwise interfere with recycling of lipid I in stage II peptidoglycan biosynthesis (Table 1).

**Table 1:** **Non-inducers of liaI Expression and Inducers of liaI Expression**.

**Compound**	**liaI Expression**	**Disk diffusion assay response**
Kanamycin	Non-inducer	Negative
Rifampicin	Non-inducer	Negative
Tetracycline	Non-inducer	Negative
Chloramphenicol	Non-inducer	Negative
Spectinomycin	Non-inducer	Negative
Streptomycin	Non-inducer	Negative
SDS	Non-inducer	Negative
Triton	Non-inducer	Negative
DNP	Non-inducer	Negative
Lysozyme	Non-inducer	Negative
Ampicillin	Inducer	Negative
Bacitracin	Inducer	Positive
Cepaholsporin	Inducer	Negative
D-cycloserine	Inducer	Negative
Moenomycin	Inducer	Negative
Nisin	Inducer	Positive
Penicillin G	Inducer	negative
Polymyxin B	Inducer	negative
Ramoplanin	Inducer	Positive
Tunicamycin	Inducer	Positive
Vancomycin	Inducer	Positive

*Activity was assessed by the appearance of a blue ring around the edge of the zone of inhibition on LB agar plates supplemented with X-Gal. (Compounds were tested in a range concentration of 50–0.5 mg/mL)*.

*B. subtilis* strain, HB0953, contains the lacZ fusion but additionally has a kanamycin insertion into the liaR gene which inactivates liaR. Since an intact two-component liaRS system is required for induction of the liaIH operon, inactivation of this system by kan insertion into liaR results in the inability of true inducers of P liaI to turn on β-galactosidase production. On X-gal medium, the LiaRS response appears to be very specific for true inducers and no false positive responses on HB0950 have been observed. However, with S-gal, fluoroquinolones, and cephalosporins produce a response on HB0950 that might be interpreted as a weak positive. These can be differentiated from true positives through the use of HB0953. On HB0953, true positives such as bacitracin and ramoplanin give an unambiguously negative response, while fluoroquinolones and cephalosporins give the same weak positive response as they do on HB0950.

### Natural products screening for lipid II inhibitors

**Figure 2:**
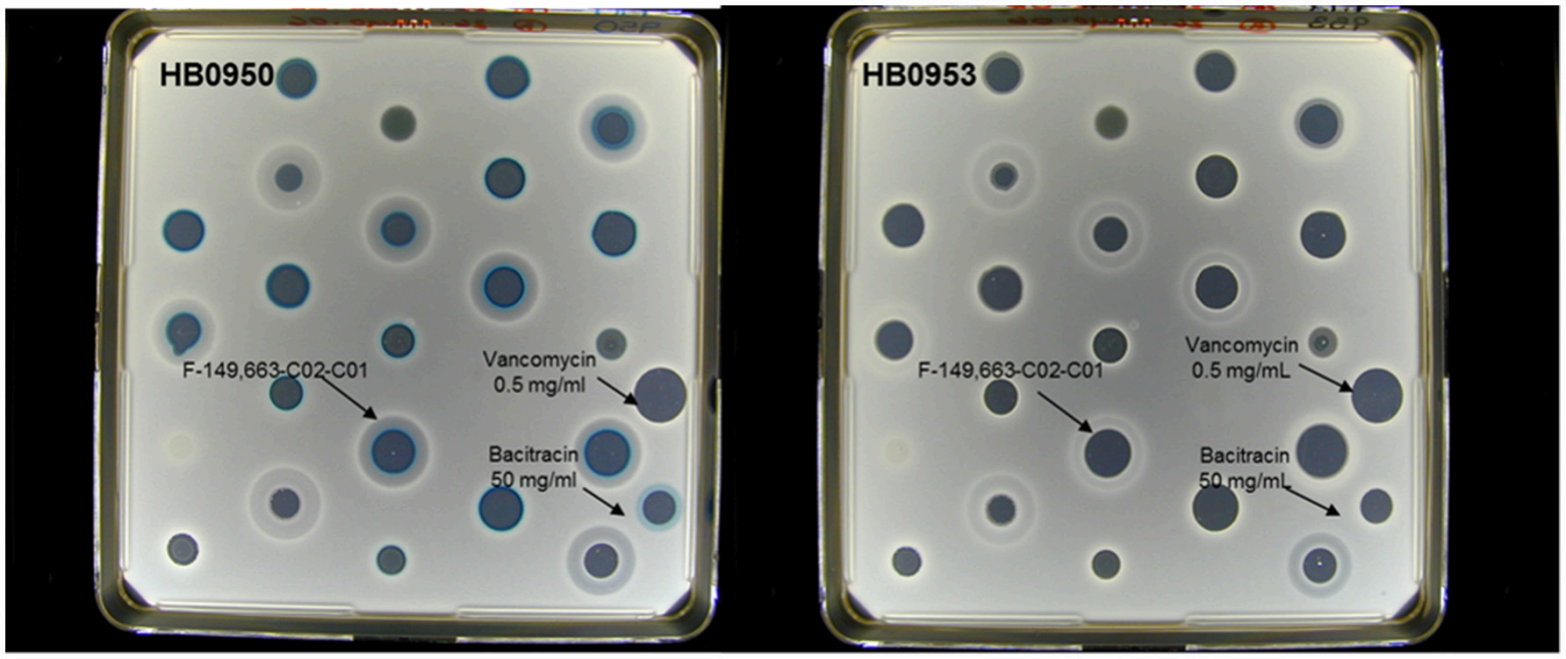
**Example of the response of some active (blue ring response) extracts in the agar- based LiaRS assay (Nunc-plate)**.

A set of 37,000 natural products (NP) extracts (50% bacteria and 50% fungi) were screened against the LiaRS assay described above at a single concentration (1xWBE), giving a hit rate of 0.13%. As controls, extracts with bacitracin and vancomycin were applied in each plate onto the agar surface (Figure 2). Extracts that caused induction of β-galactosidase in *B. subtilis* strain HB0950 were tested for specificity by re-testing them against both strains HB0950 and HB0953. The blue response was rated from 1 to 3 according to their intensity. A total of 49 actinomycetes strains were authentic inducers of LiaRS activity causing a blue response against HB0950 but not against HB0953. The larger and bluer inhibition zones were produced by a series of extracts within the group of actinomycetes.

### Characterization of actinomycete strains

The soil samples used for the isolation of the 49 actinomycete strains were collected worldwide comprising both tropical and temperate areas: Costa Rica, French Guyana, Mexico, New Caledonia, South Africa, Spain, and Sri Lanka, including different ecological habitats like agricultural soils, riverbeds, lakes, ponds, swamps, dunes, tropical and sempervirent forests, savanna soil, and rhizospheres.

The most abundant taxonomic group identified by morphological analysis was the family *Micromonosporaceae* (69.3%, 34 strains most in the genera *Micromonospora*). Also, 3 strains were assigned to *Nocardia*, 3 strains to *Streptomyces*, 1 strain to *Actinomadura*, 2 strains to *Amycolatopsis*, and 1 strain to *Actinoplanes*. A set of 5 strains could not be identified (Table 2).

**Table 2:** **Taxonomy and number of strains isolated from both tropical and temperate countries**.

**Strain**	**Origin**	**Ecological habitats**	**Family**	**Closest neighbor (EzTaxon result)**	**Similarity**	**Accession number**
F-178,252	Mexico	Soil	Micromonosporaceae	Actinoplanes ferrugineus	97.98	KY454560
F-151,217	French Guyana	Rainforest		Micromonospora chaiyaphumensis	99.35	KY454536
F-151,215			Micromonospora sp. (^*^)			
F-175,917	Mexico	Pond		Micromonospora chaiyaphumensis	99.06	KY454552
F-179,413			Micromonospora chaiyaphumensis	99.85		KY454568
F-143,677	New Caledonia	Savannah		Micromonospora chaiyaphumensis	99.78	KY454532
F-182,202	South Africa	Rhizosphere		Micromonospora chaiyaphumensis	99.64	KY454570
F-182,239			Micromonospora chaiyaphumensis	99.64		KY454571
F-182,241			Micromonospora chaiyaphumensis	99.64		KY454572
F-188,416			Micromonospora chaiyaphumensis	99.64		KY454573
F-188,417			Micromonospora chaiyaphumensis	99.64		KY454574
F-188,442			Micromonospora chaiyaphumensis	99.63		KY454575
F-162,175	French Guyana	Rainforest		Micromonospora chersina	99.64	KY454541
F-140,177	New Caledonia	Sempervirent forest		Micromonospora sp. (^*^)		
F-144,426			Micromonospora chersina	99.64		KY454533
F-149,663		Savannah	Micromonospora chersina		99.72	KY454535
F-143,676			Micromonospora sp. (^*^)			
F-169,627	Sri Lanka	Mangrove		Micromonospora chersina	99.71	KY454545
F-175,357			Micromonospora chersina	99.64		KY454551
F-177,720			Micromonospora chersina	99.78		KY454556
F-177,770			Micromonospora chersina	99.57		KY454557
F-177,777			Micromonospora chersina	99.71		KY454558
F-178,645			Micromonospora chersina	99.71		KY454563
F-178,647			Micromonospora chersina	99.71		KY454564
F-179,454			Micromonospora chersina	99.64		KY454569
F-151,222	French Guyana	Rainforest		Micromonospora endolithica	99.57	KY454537
F-170,297	South Africa	Rhizosphere		Micromonospora equina	99.41	KY454546
F-178,247	Mexico	Soil		Micromonospora fulviviridis	99.42	KY454559
F-179,400		Pond	Micromonospora halotolerans		99.64	KY454567
F-161,233	South Africa	Dune		Micromonospora halotolerans	99.85	KY454540
F-168,651		Rhizosphere	Micromonospora halotolerans		99.64	KY454542
F-169,254			Micromonospora inositola	99.43		KY454543
F-169,257			Micromonospora inositola	99.48		KY454544
F-170,386			Micromonospora inositola	99.5		KY454547
F-171,509			Micromonospora inositola	99.49		KY454548
F-146,703	New Caledonia	Sempervirent forest	Nocardiaceae	Nocardia niigatensis	99.86	KY454534
F-161,197	South Africa	Dune		Nocardia testacea	99.64	KY454539
F-186,787		Rhizosphere	Nocardia sp. (^*^)			
F-117,410	Costa Rica	Agricultural land	Pseudonocardiaceae	Amycolatopsis umgeniensis	99.71	KY454530
F-178,253	Mexico	Soil		Lechevalieria atacamensis	98.99	KY454561
F-177,328		Lake	Streptomycetaceae	Streptomyces atrovirens	99.62	KY454553
F-177,430			Soil	Streptomyces atrovirens	99.57	KY454554
F-152,578	Spain		River bed	Streptomyces mirabilis	99.36	KY454538
F-151,073	French Guyana	Rainforest	Thermomonosporaceae	Actinomadura sp. (^*^)		
F-175,914	Mexico	Pond	Unidentified	Unidentified (^*^)		
F-161,198	South Africa	Dune		Unidentified (^*^)		
F-161,203			Unidentified (^*^)			
F-161,272			Unidentified (^*^)			
F-171,173		Rhizosphere	Unidentified (^*^)			

(*) *16S rDNA gene sequence not available*.

### Evaluation of antimicrobial activity

Most of the extracts exhibited activity against Gram-positive bacteria (*S. aureus, Enterococcus faecalis*). Their IC_90_-values oscillated in a range of 0.06–0.015 WBE/mL, together with some extracts which showed a IC_90_ value below 0.002 WBE/mL. Contrary, none of the extracts displayed activity against Gram-negative bacteria (*Haemophilus influenzae, Pseudomonas aeruginosa, Escherichia coli*) or against *Aspergillus fumigatus*, nor against *Candida albicans*. They showed MIC-values above the first point of dilution (>0.06 WBE/mL) except for extract F-144,426-C03-C01 that showed weak antifungal activity against *C. albicans* giving a value of 0.06 WBE/mL (Table 3).

**Table 3:** **Extracts tested in the LiaRS assay, in the susceptibility testing assays, and for High Resolution MS analysis**.

**Strain**	**Extract**	**IC_90_ WBE/mL**								***A. fumigatus***		***LiaRS_Assay***
		^a^***Medium (days)***	***S. aureus***	***S. aureus***	***H. influenzae***	***E. faecalis***	***C. albicans***	***MF_5668***		***HB950***	***HB953***	
			***CL_8260***	**EPI_167**	***CLB21526***	***ATCC_29212***	***MY_1055***	**ZOI (mm)|Q**		**ZOI (mm)|Q(#)**	**ZOI (mm)|Q**	***LC-MS***
F-117,410	F-117,410-C01-C01	CLA(13)	(−)	(−)	(−)	(−)	(−)	0		7|C**(2)**	7|C	Related to ramoplanin
F-140,177	F-140,177-C01-C01	CLA(13)	(−)	(−)	(−)	(−)	(−)	0		8|C**(2)**	8|C	Related to ramoplanin
F-143,676	F-143,676-C02-C01	FR23(13)	0.06	0.06	(−)	0.06	(−)	0		9|B**(2)**	8|B	Related to ramoplanin
F-143,677	F-143,677-C02-C01	FR23(13)	(−)	(−)	(−)	(−)	(−)	0		8|C**(2)**	8|C	Related to ramoplanin
F-144,426	F-144,426-C03-C01	GOT(13)	0.06	0.06	(−)	0.06	0.06	11|A		14A**(2)**	14A	Related to ramoplanin
F-146,703	F-146,703-C02-C01	GOT(7)	(−)	(−)	(−)	(−)	(−)	0		9|A**(3)**	11|A	Related to ramoplanin
	F-146,703-C03-C01	MPG(7)	(−)	(−)	(−)	0.06	(−)	0		13|A**(3)**	15|A	Related to ramoplanin
F-149,663	F-149,663-C01-C01	CLA(13)	0.06	0.06	(−)	0.06	(−)	0		16|A**(3)**	17|A	Related to ramoplanin
	F-149,663-C02-C01	GOT(13)	(−)	0.06	(−)	0.06	(−)	0		17A**(3)**	19A	Related to ramoplanin
	F-149,663-C03-C01	MPG(13)	(−)	(−)	(−)	(−)	(−)	0		11|A**(3)**	11|A	Related to ramoplanin
F-151,073	F-151,073-C01-C01	FR23(13)	0.06	0.06	(−)	(−)	(−)	0		8|B**(2)**	8|B	Related to ramoplanin
F-151,215	F-151,215-C01-C01	FR23(13)	(−)	(−)	(−)	(−)	(−)	0		7|B**(2)**	8|B	Related to ramoplanin
F-151,217	F-151,217-C02-C01	GOT(13)	(−)	(−)	(−)	(−)	(−)	0		27|D**(2)**	26|D	Related to ramoplanin
F-151,222	F-151,222-C01-C01	FR23(13)	0.015	0.03	(−)	0.015	(−)	0		16|A**(3)**	18|A	A2
	F-151,222-C02-C01	GOT(13)	(−)	(−)	(−)	(−)	(−)	0		16|A**(3)**	15|A	A2
F-152,578	F-152,578-C01-C01	DNPM(7)	(−)	(−)	(−)	(−)	(−)	0		7|C**(2)**	7|C	Related to ramoplanin
F-161,197	F-161,197-C01-C01	GOT(13)	(−)	(−)	(−)	(−)	(−)	27|E		27|D**(2)**	27|D	Related to ramoplanin
	F-161,197-C03-C01	RAM2(13)	(−)	(−)	(−)	(−)	(−)	0		7|E**(2)**	7|E	Related to ramoplanin
F-161,198	F-161,198-C02-C01	MPG(13)	0.06	0.06	(−)	0.06	(−)	0		10|A**(2)**	10|A	Related to ramoplanin
F-161,203	F-161,203-C01-C01	GOT(13)	0.06	0.06	(−)	(−)	(−)	0		11|A**(2)**	10|A	Related to ramoplanin
F-161,233	F-161,233-C01-C01	GOT(13)	0.06	0.06	(−)	0.03	(−)	0		14|A**(3)**	16|A	Related to ramoplanin
F-161,272	F-161,272-C01-C01	GOT(13)	(−)	0.06	(−)	0.06	(−)	0		8|B**(2)**	8|B	Related to ramoplanin
F-162,175	F-162,175-C03-C01	MPG(13)	0.06	0.015	(−)	0.03	(−)	0		15|A**(3)**	16|A	Related to ramoplanin
F-168,651	F-168,651-C02-C01	GOT(13)	0.06	0.03	(−)	0.06	(−)	0		16|A**(3)**	18|A	Related to ramoplanin
	F-168,651-C03-C01	MPG(13)	(−)	0.06	(−)	0.03	(−)	0		15|A**(3)**	15|A	Related to ramoplanin
F-169,254	F-169,254-C02-C01	GOT(13)	(−)	(−)	(−)	0.06	(−)	0		16|A**(3)**	17|A	Related to ramoplanin
	F-169,254-C03-C01	MPG(13)	(−)	(−)	(−)	(−)	(−)	0		11|A**(3)**	11|A	Related to ramoplanin
F-169,257	F-169,257-C02-C01	GOT(13)	(−)	(−)	(−)	(−)	(−)	0		12|A**(3)**	13|A	Related to ramoplanin
	F-169,257-C03-C01	MPG(13)	0.06	0.06	(−)	0.06	(−)	0		13|A**(3)**	13|A	Related to ramoplanin
F-169,627	F-169,627-C01-C01	FR23(13)	(−)	(−)	(−)	(−)	(−)	0		14|C**(2)**	13|C	Related to ramoplanin
F-170,297	F-170,297-C02-C01	GOT(13)	0.06	0.06	(−)	(−)	(−)	0		12|A**(2)**	11|A	Related to ramoplanin
F-170,386	F-170,386-C01-C01	FR23(13)	(−)	0.03	(−)	0.06	(−)	0		13|A**(3)**	13|A	Related to ramoplanin
	F-170,386-C02-C01	GOT(13)	(−)	(−)	(−)	(−)	(−)	0		17|A**(3)**	19|A	Related to ramoplanin
	F-170,386-C03-C01	MPG(13)	(−)	0.06	(−)	0.03	(−)	0		14|A**(3)**	16|A	Related to ramoplanin
F-171,173	F-171,173-C02-C01	GOT(13)	0.06	0.06	(−)	0.06	(−)	0		9|B**(2)**	9B	Related to ramoplanin
F-171,509	F-171,509-C01-C01	FR23(13)	(−)	(−)	(−)	(−)	(−)	0		12|A**(3)**	13|A	Related to ramoplanin
	F-171,509-C02-C01	GOT(13)	(−)	(−)	(−)	(−)	(−)	0		11|C**(2)**	11|C	Related to ramoplanin
F-175,357	F-175,357-C03-C01	SOTM(13)	0.0075	0.001875	0.06	0.0075	(−)	0		13|A**(2)**	12A	Related to ramoplanin
F-175,914	F-175,914-C02-C01	FR23(13)	(−)	0.06	(−)	(−)	(−)	0		7|C(2)	7|C	Related to ramoplanin
F-175,917	F-175,917-C02-C01	FR23(13)	(−)	0.06	(−)	(−)	(−)	0		7|C**(2)**	7|C	Related to ramoplanin
F-177,328	F-177,328-C02-C01	MPG(7)	0.03	0.015	(−)	0.03	(−)	0		17|A**(3)**	20|A	Related to ramoplanin
F-177,430	F-177,430-C02-C01	MPG(7)	0.0075	0.00375	(−)	0.015	(−)	0		19|A**(3)**	21|A	Related to ramoplanin
F-177,720	F-177,720-C01-C01	CLA(13)	(−)	(−)	(−)	(−)	(−)	0		12|A**(3)**	13|A	Related to ramoplanin
	F-177,720-C02-C01	FR23(13)	(−)	0.03	(−)	0.06	(−)	0		14|A**(3)**	13|A	A2/A1
F-177,770	F-177,770-C01-C01	CLA(13)	(−)	(−)	(−)	(−)	(−)	(−)	0	14|A**(3)**	16|A	A2/A1
	F-177,770-C02-C01	FR23(13)	0.001875	0.001875	0.06	0.00375	(−)	(−)	0	14|A**(2)**	14|A	related to ramoplanin
F-177,777	F-177,777-C02-C01	FR23(13)	0.015	0.015	(−)	0.015	(−)	(−)	0	14|A**(3)**	14|A	Related to ramoplanin
F-178,247	F-178,247-C02-C01	FR23(13)	(−)	(−)	(−)	(−)	(−)	(−)	0	6|B**(2)**	6|B	Related to ramoplanin
F-178,252	F-178,252-C01-C01	CLA(13)	(−)	(−)	(−)	(−)	(−)	(−)	0	12|A**(2)**	13|A	Related to ramoplanin
F-178,253	F-178,253-C01-C01	CLA(13)	(−)	(−)	(−)	(−)	(−)	(−)	0	14|E**(2)**	14|E	Related to ramoplanin
F-178,645	F-178,645-C01-C01	CLA(13)	(−)	(−)	(−)	(−)	(−)	(−)	0	13|A**(3)**	14|A	Related to ramoplanin
	F-178,645-C02-C01	FR23(13)	0.03	0.03	(−)	0.06	(−)	(−)	0	13|A**(3)**	14|A	Related to ramoplanin
F-178,647	F-178,647-C01-C01	CLA(13)	0.06	0.015	(−)	0.06	(−)	(−)	0	13|A**(3)**	15|A	A2
	F-178,647-C02-C01	FR23(13)	0.015	0.015	(−)	0.03	(−)	(−)	0	16|A**(3)**	17|A	A1/A2
F-179,400	F-179,400-C01-C01	CLA(13)	(−)	(−)	(−)	(−)	(−)	(−)	0	10|A**(3)**	11|A	Related to ramoplanin
F-179,413	F-179,413-C01-C01	CLA(13)	(−)	(−)	(−)	0.06	(−)	(−)	0	18|A**(3)**	19|A	Related to ramoplanin
	F-179,413-C02-C01	FR23(13)	(−)	0.06	(−)	0.06	(−)	(−)	0	15|A**(3)**	16|A	Related to ramoplanin
F-179,454	F-179,454-C02-C01	FR23(13)	0.015	0.0075	(−)	0.03	(−)	(−)	0	13|A**(3)**	13|A	Related to ramoplanin
F-182,202	F-182,202-C03-C01	MPG(13)	0.03	0.03	(−)	0.06	(−)	(−)	0	11|A**(2)**	11|A	Related to ramoplanin
F-182,239	F-182,239-C01-C01	FR23(13)	(−)	(−)	(−)	(−)	(−)	(−)	0	12|A**(2)**	12|A	Related to ramoplanin
F-182,241	F-182,241-C01-C01	FR23(13)	(−)	0.06	(−)	0.06	(−)	(−)	0	13|A**(3)**	13|A	Related to ramoplanin
	F-182,241-C03-C01	MPG(13)	(−)	0.06	(−)	0.06	(−)	(−)	0	11|A**(3)**	13|A	Related to ramoplanin
F-186,787	F-186,787-C01-C01	CLA(7)	(−)	0.06	(−)	0.06	(−)	(−)	0	6|B**(2)**	6|B	Related to ramoplanin
F-188,416	F-188,416-C01-C01	FR23(13)	(−)	(−)	(−)	(−)	(−)	(−)	0	9|C**(2)**	9|C	Related to ramoplanin
F-188,417	F-188,417-C01-C01	FR23(13)	0.03	0.015	(−)	0.015	(−)	(−)	0	14|A**(3)**	14|A	Related to ramoplanin
F-188,442	F-188,442-C01-C01	FR23(13)	(−)	(−)	(−)	(−)	(−)	(−)	0	9|C**(2)**	9|A	Related to ramoplanin

*ZOI (mm), Halo of inhibition*.

*Q, Qualifier (A or B-clear, C or D-hazy, E-very hazy)*.

*(#) β-galactosidase intensity (blue ring)*.

*Ramoplanin analogs: A1 MW = 2538; A2 MW = 2552; A3 MW = 2566*.

*IC90 > 0.06: (−)*.

a *Media and days of cultivation. Media composition are indicated in Supplementary Data*.

These results indicate that the extracts keep similar antibacterial spectrum to ramoplanins.

### Inhibition peptidoglycan biosynthesis

**Figure 3:**
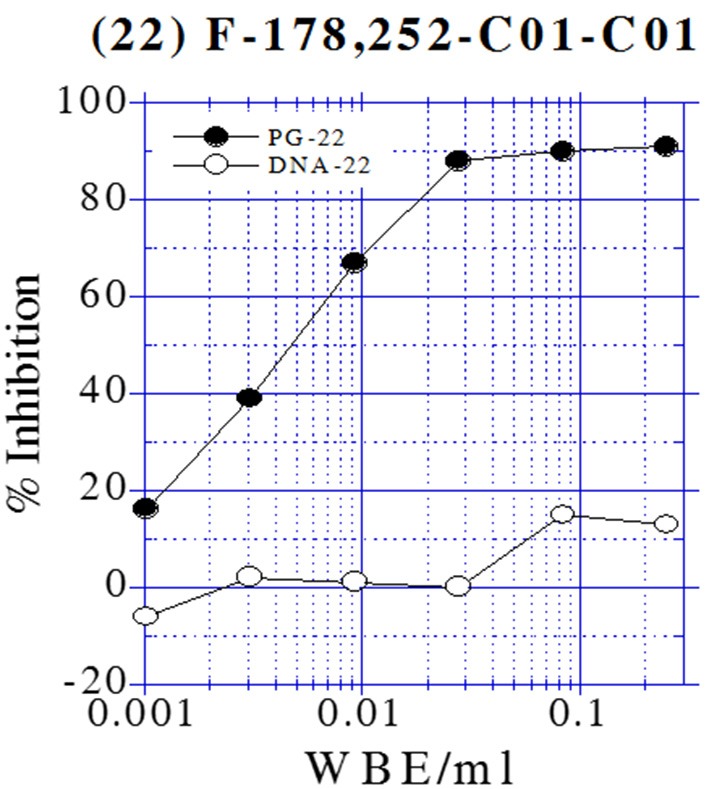
**Dose-Response curves from the Labeling test of one of the active extracts from the NP screening in *B. subtilis* HB0950**.

All extracts tested for inhibition of macromolecular labeling exhibited specific activity by inhibiting peptidoglycan synthesis (PG) above 80%. Figure 3 illustrates an example of the labeling test of one of the active extracts from the NP screening in a two-fold serial dilution series for inhibition of DNA and peptidoglycan syntheses in *B. subtilis* HB0950. This sample inhibited PG to a greater extent than they inhibited protein or DNA syntheses, indicating that it is a selective inhibitor of PG synthesis.

### Chemical analysis of LiaRS positive extracts

**Figure 4:**
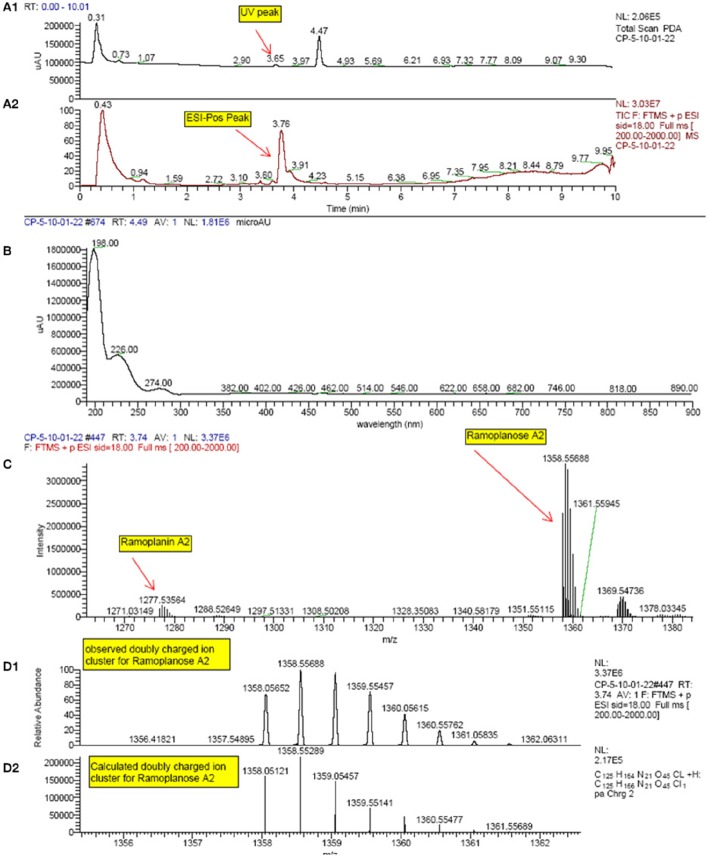
**HPLC and High Resolution LC-MS spectrum profile of some of the actives from the natural products screening which matched with Ramoplanin A2 and Ramoplanose**. (Extract example: F-151,222-C01-C01). **(A1)**: UV-visible trace; **(A2)**: TIC (Total ion chromatogram); **(B)**: UV-visible spectra at 4.49 min; **(C)**: Mass spectra at 3.74 min from FTMS positive ion; **(D1)**: Mass spectra at 3.74 min. **(D2)**: Theoretical isotopic distribution of Ramoplanose A2.

All extracts that were confirmed hits in the LiaRS screen were analyzed by HR-LCMS. Since ramoplanin was a known compound active in this assay, sample dereplication was performed in order to eliminate those samples containing this molecule from undergoing further isolation efforts. High resolution mass spectrometry signatures of each extract were evaluated and scanned for single ions corresponding to doubly charged species of ramoplanin A1, A2, and A3. In addition, the presence of ramoplanoses A1, A2, and A3, glycosylated versions of the ramoplanins, was also observed (Figure 4). Further, some additional ramoplanin analogs were also identified. These analogs corresponded to reduced versions (+4H and +O,4H) of ramoplanins, with the modifications presumably occurring in the N-acyl hydrophobic sidechain. All extracts showed mass spectrometry signatures (range of molecular weights and isotopic distributions) that confirmed the presence of compounds related to ramoplanin structural family and within them new analog compounds.

## Discussion

Ramoplanin is a suitable clinical candidate for the treatment of infections caused by aerobic or anaerobic Gram-positive pathogens (Farver et al., 2005). The mechanism of action of ramoplanin makes it unlikely that it will develop high levels of resistance since this compound acts on the second phase of peptidoglycan biosynthesis, capturing the Lipid intermediate II (Somner and Reynolds, 1990).

Thus, far, ramoplanin was only isolated from the fermentation of a strain of *Actinoplanes* sp. ATCC 33076 (Farver et al., 2005). However, in this study we have found 49 distinct actinomycete strains producing compounds related to this antibiotic. Further, our findings demonstrate that the extracts prepared from this group of strains display antibacterial profiles similar to ramoplanin.

These extracts revealed a positive response to the agar diffusion assay developed from studies in Cornell University (Mascher et al., 2003, 2004) showing activity against *B. subtilis* strains HB0950 and HB0953, being that they revealed a blue ring (X-Gal hydrolysis by the presence of β-galactosidase) in the strain containing the P liaI-lacZ fusion and they did not show such a response in the strain that additionally contained a gene insertion of kanamycin resistance in the *liaR* gene. Extracts positive in this test proved to be compounds that specifically interfere with the Lipid II as ramoplanin does (Mascher et al., 2004).

Moreover, most of them exhibited specific activities against Gram-positive bacteria (*S. aureus, E. faecalis*), as shown in Table 3. Also, all the extracts completely and selectively inhibited peptidoglycan biosynthesis, as demonstrated by the results obtained in a macromolecular labeling assay.

Analysis of the extracts by HR-LCMS indicated that they all contained compounds that belong to ramoplanin family. Dereplication was able to quickly identify these components and eliminated the need for extensive isolation efforts. Scale-up of selected cultures and isolation of novel ramoplanin analogs identified is in progress.

The point that most of the producing organisms belong to the *Micromonosporaceae* family is consistent with the taxonomy of the original organism producer of ramoplanin (Farver et al., 2005). Our results additionally suggest that the production of ramoplanin analogs is widespread within the actinomycetes. Geographically, the producers of these compounds were dispersed throughout the whole world based on the different origin of the samples from which the strains of our study came from.

The results obtained in this study support the fact that natural products are an unlimited source of potential drugs, in particular of antibiotics (Peláez, 2006; Chopra, 2013; Lacret et al., 2015; Ling et al., 2015; Crespo et al., 2016). They also demonstrate the power of using novel screening strategies that combine new knowledge in biotechnology with libraries of natural products in order to find new drug candidates against multi-drug resistance pathogens.

Our data strongly suggest the presence of new ramoplanin-analogs among the actinomycete strains of this study, so large scale fermentations and purifications of selected strains are being currently performed in order to identify these new antimicrobials which perhaps could overcome the low local tolerability of ramoplanin when injected intravenously.

## Authors contributions

MD performed the HTS assay, collected, and analyzed data; IG performed the fermentation of microorganisms and taxonomic identification of the microbial strains. OG supervised the microbial work; CP, DZ, JM performed the chemical work; RO performed macromolecular labeling assay; JT, NE generated the extracts; FP general supervision; MD, CP, FR, FV, and OG wrote the manuscript which was revised and approved by all the authors.

### Conflict of interest statement

The authors declare that the research was conducted in the absence of any commercial or financial relationships that could be construed as a potential conflict of interest.
