# Supplementary material for: Production of Ramoplanin and Ramoplanin Analogs by Actinomycetes
Source: Front Microbiol. 2017 Mar 6;8:343. doi: 10.3389/fmicb.2017.00343 (PMC5337499; doi:10.3389/fmicb.2017.00343)
Supplement: Supplementary file 2 [file Table2.docx]

|  |  |
| --- | --- |
| **CLA** |  |
|  | g/L |
| **Lactose** | 40 |
| **Corn meal** (SIGMA-C6304) | 40 |
| **Ardamine** | 5 |
| **deionized water** | **1000ml** |
| **pH 7.0** |  |
|  |  |
| **FR23** |  |
|  | g/L |
| **Glucose** (PANREAC 131341) | 5 |
| **soluble starch from potato** (PANREAC 121096) | 30 |
| **cane molasses** | 20 |
| **Pharmamedia** | 20 |
| **deionized water** | **1000ml** |
| **pH 7.0** |  |
|  |  |
|  |  |
| **DNPM** |  |
|  | g/L |
| **Dextrin from corn** **Type I** (SIGMA D2006) | 40 |
| **N-Z Soy BL** (SIGMA P6713) | 7.5 |
| **Primary yeast** | 5 |
| **MOPS** (CALBIOCHEM 475898) | 21 |
| **deionized water** | **1000ml** |
| pH 7.0 |  |
|  |  |
|  |  |
| **GOT** |  |
|  | g/L |
| **Glycerol** (PANREAC 141339) | 60 |
| **Oat flour** | 15 |
| **Tomato paste** | 5 |
| **CO3Ca** (añadir después de pH) (MERCK 8605747) | 3 |
| **deionized water** | **1000ml** |
| **pH 7.0** |  |
|  |  |
|  |  |
|  |  |
| **MPG** |  |
|  | g/L |
| **Glucose** (PANREAC 131341) | 10 |
| **Millet meal** | 20 |
| **Pharmamedia** | 20 |
| **MOPS** | 20 |
| **deionized water** | **1000ml** |
| **pH 7.0** |  |
|  |  |
|  |  |
| **RAM2** |  |
|  | g/L |
| **Glucose** (PANREAC 131341) | 10 |
| **Maltose** (MERCK 1.05910.0500) | 15 |
| **Corn meal yellow** (SIGMA C6704) | 4 |
| **primary yeast** | 5 |
| **Pharmamedia** | 7.5 |
| **deionized water** | **1000ml** |
| pH 7.0 |  |
|  |  |
|  |  |
| **SOTM** |  |
|  | **g/l** |
| **Sucrose** | 45 |
| **MOPS** | 21 |
| **Oat flour** | 15 |
| **Tomate paste** | 5 |
| **CO3Ca** | 3 |
|  |  |
